# Anti-tumor activity of cetuximab plus avelumab in non-small cell lung cancer patients involves innate immunity activation: findings from the CAVE-Lung trial

**DOI:** 10.1186/s13046-022-02332-2

**Published:** 2022-03-26

**Authors:** Carminia Maria Della Corte, Morena Fasano, Vincenza Ciaramella, Flora Cimmino, Robert Cardnell, Carl M. Gay, Kavya Ramkumar, Lixia Diao, Raimondo Di Liello, Giuseppe Viscardi, Vincenzo Famiglietti, Davide Ciardiello, Giulia Martini, Stefania Napolitano, Concetta Tuccillo, Teresa Troiani, Erika Martinelli, Jing Wang, Lauren Byers, Floriana Morgillo, Fortunato Ciardiello

**Affiliations:** 1 Medical Oncology, Department of Precision Medicine, Università degli Studi della Campania “Luigi Vanvitelli”, Via S. Pansini 5, 80131 Naples, Italy; 2 CEINGE Institute, Naples, Italy; 3 Department of Thoracic Head and Neck Medical Oncology, University of Texas, MD Anderson Cancer Center, Houston, TX USA; 4 Department of Bioinformatics & Computational Biology, The University of Texas MD Anderson Cancer Center, Texas Houston, USA

**Keywords:** Innate immunity, STING, NK cells, Cetuximab, Avelumab

## Abstract

**Background:**

We recently conducted Cetuximab-AVElumab-Lung (CAVE-Lung), a proof-of-concept, translational and clinical trial, to evaluate the combination of two IgG1 monoclonal antibodies (mAb): avelumab, an anti-PD-L1 drug, and cetuximab, an anti-epidermal growth factor receptor (EGFR) drug, as second- or third-line treatment in non-small cell lung cancer (NSCLC) patients. We have reported clinically relevant anti-tumor activity in 6/16 patients. Clinical benefit was accompanied by Natural Killer (NK) cell-mediated antibody-dependent cell cytotoxicity (ADCC). Among the 6 responding patients, 3 had progressed after initial response to a previous treatment with single agent anti-PD-1, nivolumab or pembrolizumab.

**Methods:**

We report long-term clinical follow-up and additional findings on the anti-tumor activity and on the immune effects of cetuximab plus avelumab treatment for these 3 patients.

**Results:**

As of November 30, 2021, 2/3 patients were alive. One patient was still on treatment from 34 months, while the other two patients had progression free survival (PFS) of 15 and 19 months, respectively. Analysis of serially collected peripheral blood mononuclear cells (PBMC) revealed long-term activation of NK cell-mediated ADCC. Comprehensive genomic profile analysis found somatic mutations and germline rare variants in DNA damage response (DDR) genes. Furthermore, by transcriptomic analysis of The Cancer Genome Atlas (TCGA) dataset we found that DDR mutant NSCLC displayed high STING pathway gene expression. In NSCLC patient-derived three-dimensional in vitro spheroid cultures, cetuximab plus avelumab treatment induced additive cancer cell growth inhibition as compared to single agent treatment. This effect was partially blocked by treatment with an anti-CD16 mAb, suggesting a direct involvement of NK cell activation. Furthermore, cetuximab plus avelumab treatment induced 10-, 20-, and 20-fold increase, respectively, in the gene expression of *CCL5* and *CXCL10*, two STING downstream effector cytokines, and of *interferon β*, as compared to untreated control samples.

**Conclusions:**

DDR mutations may contribute to DDR-induced STING pathway with sustained innate immunity activation following cetuximab plus avelumab combination in previously treated, PD-1 inhibitor responsive NSCLC patients.

**Supplementary Information:**

The online version contains supplementary material available at 10.1186/s13046-022-02332-2.

## Background

Immunotherapy with immune checkpoint inhibitors has completely reshaped the standard of care for several cancer types, including non-small cell lung cancer (NSCLC) [1]. According to international guidelines, advanced/metastatic NSCLC patients should receive treatment with anti-PD-1/PD-L1 (Programmed-Death−/PD-Ligand 1) drugs as single agent or in combination with chemotherapy, based on PD-L1 tumor expression [2]. However, therapeutic efficacy and duration of response are very heterogenous [3]. Among others, an open question is if rechallenge with novel combinations including immunotherapy may be effective in NSCLC patients that have progressed after initial response to immunotherapy [4].

We have recently conducted a proof-of-concept study in pre-treated NSCLC patients with the combination of the anti-epidermal growth factor receptor (EGFR) monoclonal antibody (mAb) cetuximab and the anti-PD-L1 mAb avelumab (the Cetuximab-AVElumab-Lung, CAVE-Lung trial) [5]. Neither avelumab or cetuximab are currently approved for NSCLC, but they showed promising activity in clinical trials for PD-L1 high- [6] or EGFR high-expressing NSCLC patients, respectively [7, 8]. In the CAVE-Lung trial, 16 pre-treated NSCLC patients were enrolled, without selection for PD-L1 tumor expression or any other biomarker. We observed clinical activity of cetuximab plus avelumab in 6 patients, that experienced progression free survival (PFS) of 8 months or more [5]. In these 6 patients, tumors had *EGFR* wild-type gene with variable levels of PD-L1 protein expression. Three out of 16 patients had received immunotherapy before entering the CAVE-Lung trial; having progressed, after an initial response, to previous single agent therapy with pembrolizumab or nivolumab. These 3 patients obtained a significant clinical benefit by the rechallenge of immunotherapy with an anti-PD-L1 drug (avelumab) in combination with an anti-EGFR drug (cetuximab).

The rationale for the synergistic anti-tumor activity of cetuximab plus avelumab could be based on a double mechanism, such as the direct inhibition of two biologically relevant targets for NSCLC, EGFR and PD-L1 [6–8] as well as the ability of these two IgG1 mAbs to bind natural killer (NK) cell FC receptors (FCG3A/CD16) and to induce antibody-dependent cell cytotoxicity (ADCC) [9].

NK cell activation represents an innate antigen-independent immune response and is supposed to act together with adaptive T-cell antigen-driven immunity [9]. However, how this interplay between innate and adaptive immunity takes place in the host response to cancer has not yet fully understood. Among various players for eliciting innate immune responses, the stimulator of interferon genes (STING) pathway is physiologically activated by cytosolic DNA to induce type I interferon (IFN)-driven inflammatory response [10]. In cancer cells, the STING pathway may be activated intrinsically by cytosolic DNA fragmentation prompted by malignant proliferation in the presence of DNA damage response (DDR) gene alterations, or extrinsically by DNA damage induced by chemotherapy or by radiotherapy [10–13]. The final effect of STING pathway activation is to awake CD8+ T cell-mediated anti-tumor immune responses: directly, by inducing the production of two T cell-recruiting cytokines, CCL5 and CXCL10, and, indirectly, through NK cell activation [14]. In this respect, it has been provided evidence of STING pathway activation as potential biomarker and mediator of anti-tumor immune response in NSCLC [11].

Here, we report long-term clinical follow-up and translational findings on the anti-tumor activity and on the immune effects of cetuximab plus avelumab treatment in 3 previously treated, PD-1 inhibitor responsive, NSCLC patients in the CAVE-Lung trial. We provide evidence that in these patients cetuximab plus avelumab treatment activates innate immune response, that involves both activation of NK cells and of the STING pathway.

## Methods

### Peripheral blood mononuclear cell (PBMC) isolation aNd lactate dehydrogenase (LDH) release cytotoxicity assay

PBMCs were obtained from CAVE-Lung trial patients serially during treatment with cetuximab plus avelumab and evaluated for the LDH release cytotoxicity assay, as previously reported [5].

### Next generation sequencing (NGS)

Plasma samples were collected at the time of enrollment of patients in the CAVE-Lung trial, as source of circulating tumor DNA (ctDNA) for genomic profiling of tumors. NGS analysis was performed with the Foundation Liquid platform [5]. Genomic germline DNA was extracted from PBMC using QIAmp DNA Blood Midi Kit (cod#51183). Sequencing libraries were generated using Agilent SureSelect Human All Exon kit (Agilent) following manufacturer’s recommendations and sequenced on NexSeq platform (Illumina). Sequenced reads were mapped on the human reference GRCh37/hg19 using the Burrow-Wheeler Aligner. SNVs and indels were called based on quality check processes involving the following user-definable criteria: low-complexity and repeats and segmental duplications were filtered out; quality score ≥ 20, depth ≥ 10, and AB≥0.2 for heterozygous calls; call rate ≥ 0,85. was performed. Following variant calling, rare variants were enriched by the application of three filtering steps: I) variants with MAF 1% in the gnomAD; II) variant class, including missense, protein-truncating and regulatory; mutation effects, variant results in protein truncation and predicted to be deleterious from prediction tools (SIFT, POLYPHEN-2, MUTATIONTASTER, MutationAssessor, FATHMM, and FATHMM-MKL); iii) variants classified as pathogenic, likely pathogenic or VUS from the InterVar and ClinVar database. Details are in Supplementary Table 2.

### Flow cytometry analysis

For flow cytometry (fluorescence-associated cell sorting, FACS) analysis, PBMCs were washed in staining buffer (SB) (2% fetal bovine serum, FBS, 0,1% sodium azide in phosphate-buffered saline) and, after blocking for 10 min with SB plus Ab serum 20%, were stained for 30 min with the following monoclonal antibodies: anti-CD107a, anti-TIM-3 and anti-PD-L1 (Miltenyi Biotec). Stained cells were washed two times, resuspended in SB, acquired on FACS ACCURI C6 and analyzed using ACCURI C6 software (BD Biosciences).

### Viability assay in vitro three dimensional (3D) cultures from patient tumor samples

Isolation of ex-vivo 3D cultures from the 3 NSCLC patient tumor samples was done as previously described [15, 16]. The resulting tumor spheroids were seeded in 96-well plates at the density of 1 × 1000 cells/well and were treated with the indicated drugs. Cell proliferation and cytotoxicity was measured with the MTS (3-(4,5-dimethylthiazol-2-yl)-5-(3-carboxymethoxyphenyl)-2-(4-sulfophenyl)-2H-tetrazolium) Assay Kit (Abcam), according to the manufacturer instructions. The MTS assay protocol is based on the reduction of the MTS tetrazolium compound by viable cells to generate a colored formazan dye that is soluble in cell culture media. The formazan dye is quantified by measuring the absorbance at 490–500 nm.

### Quantitative real time polymerase chain reaction (PCR)

Quantitative real time PCR. Total RNA extraction and RT-qPCR (Real Time Quantitative PCR) were performed as previously described [15]. Primer sequences were reported in Supplementary Table 1. To calculate relative gene expression in value it was used the 2-ΔCt or 2-ΔΔCt method. Nonspecific signals caused by primer dimers were excluded by dissociation curve analysis and use of non-template controls.

### Gene expression analysis from TCGA database

The Cancer Genome Atlas (TCGA) dataset include 511 lung adenocarcinoma and 501 lung squamous carcinoma, with available data for transcriptomic and genomic profiles [11]. For genomic profiling, we focused on the presence of DDR mutations, according a curated list (see Supplementary Table 2).

### Statistical analysis

Statistical analysis was performed using the Graphpad Prism software V.6.0 (Graphpad Software, San Diego, California, USA).

## Results

### Long-term follow up of NSCLC patients treated with cetuximab plus avelumab in the CAVE-lung trial

**Fig. 1:**
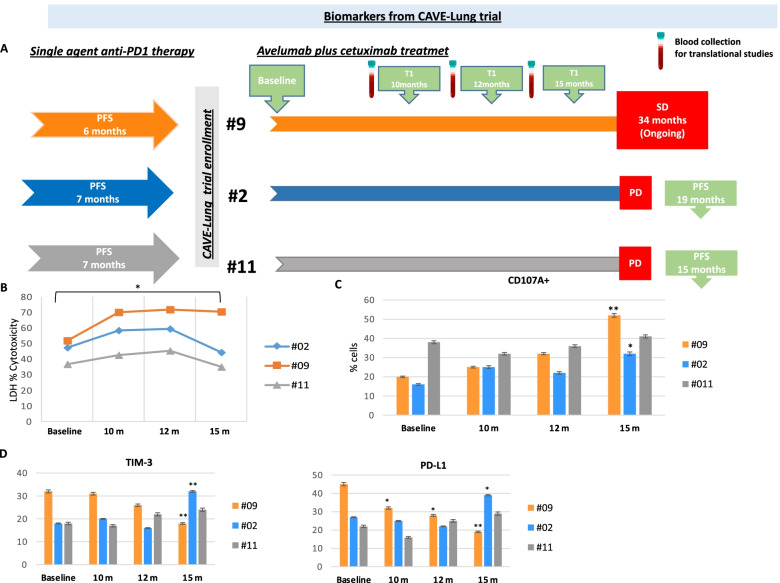
Long-term clinical and translational studies from CAVE-Lung trial patients. **A**. Among the patients enrolled in CAVE-Lung trial, 3 patients had received a previous line of therapy with single agent anti-PD-1 mAb: patient #2, pembrolizumab; patients #9 and #11, nivolumab. *Left panel.* Response to previous anti-PD-1 therapy: patient #2 obtained partial response (PR) as best radiological response with PFS of 6 months; patients #9 and #11 had stable disease (SD) with PFS of 7 and 7 months, respectively. *Right panel.* Patients were enrolled in CAVE-Lung trial and received cetuximab plus avelumab treatment, all obtaining SD as best radiologic response with PFS of 15, 19, and 34+ months, respectively. For these 3 patients, collection of PMBC during treatment for analysis of NK cell activation was done at 10, 12, and 15 months. **B**. LDH release assay in vitro was performed to assess NK cell activation from patient-derived PBMC samples. For the patient with the longest response (34 months, ongoing), LDH levels were constantly higher as compared to baseline, while in the other two patients, LDH levels were high and started to decline at the time (15 months) of progression of disease (PD) or close to PD. **C**, **D**. FACS analysis was performed to further evaluate the effects on immune cell populations, that could be induced by cetuximab plus avelumab treatment. At the time of clinical response, CD107A+ cells increased, suggesting NK cell degranulation (C); while TIM3+ and PD-L1+ immune-suppressive cells decreased, suggesting T cell activation (D). Results were inverted at the time of PD. *P*-values were calculated by ANOVA test. **p < 0.01, *p < 0.05

At 18-month longer follow-up analysis (November 30, 2021 vs April 15, 2020) [5], 3/16 patients were alive with one of these patients still on treatment (see Supplementary Fig. 1 for details). Of interest, among patients with the longest survival, 3 patients had received a previous line of therapy with single agent anti-PD-1 mAb (one patient, pembrolizumab; two patients, nivolumab); thus, suggesting clinically significant anti-tumor activity of this experimental treatment following progression to single agent immune checkpoint inhibitor therapy. These 3 patients had initial clinical benefit to previous anti-PD-1 therapy, with partial response (PR) as best radiological response in one patient and stable disease (SD) in the other two patients. PFS was 6, 7 and 7 months, respectively (Fig. 1A). Baseline tumor PD-L1 protein expression was 60, 5% and unknown, respectively. Cetuximab plus avelumab treatment obtained SD as best radiologic response in these patients with PFS of 15, 19, and 34+ months, respectively.

We have previously reported that clinical benefit following cetuximab plus avelumab treatment was accompanied by NK cells activation in the CAVE-Lung responding patients, as compared to non responders group of patients [5]. We have extended the analysis of NK cell activation in the PBMC, as assessed by LDH release cytotoxicity assay, with serial measurements during treatment at 10, 12, and 15 months, respectively, for these patients **(**Fig. 1A**)**. For the patient with the longest response (34 months, treatment ongoing), LDH levels were constantly higher as compared to baseline (increase of 20%, *p* < 0.05) while in the other two patients LDH levels were high until clinical benefit was maintained (increase of 12 and 10% respectively, *p* < 0.05) and started to decline at the time (15 months) of progression of disease (PD) or close to PD **(**Fig. 1B**)**. To further evaluate the immune effects that could be determined by cetuximab plus avelumab treatment in these patients, we analyzed by FACS the modifications in immune cells population prevalence in the PBMC. As shown in Fig. 1C, we observed an increase of CD107A+ cells, a specific marker of NK cell degranulation, at the time of clinical response: in particular, in patient #09, the one with the longest PFS, CD107A+ cells increased of 32% (*p* < 0.01) at 15 weeks and in patient #02 of 16% (*p* < 0.05). In the other patient #11 at the last time point, that was close to the time of radiological PD, we detected a not significant change CD107A+ cells (3%,p not significant). Also, we explored changes in TIM3+ and PD-L1+ immune-suppressive cells (Fig. 1 D), and we found a significant decrease of these two sub-populations at the time of clinical response in patient #09 (decrease rate of 14 and 26%, *p* < 0.01). Conversely, TIM3+ and PD-L1+ cells raised up at the time of PD in the other two patients (patient #02, increase rate of 14%, *p* < 0.01 and 12%, *p* < 0.05; patient #11, increase rate of 6 and 7%, p not significant).

Mutation profiling in ctDNA, as assessed by liquid biopsy, detected somatic mutations in DDR genes, that have been associated with a phenotype of immune responsiveness and of innate immunity activation [11]. Specifically, one patient presented *STK11 and TP53* mutations, one patient had *CHK2, ATM and BRCA2* mutations, while the third patient showed *MDM2* mutation (Fig. 1E).

Recent genetic studies suggest that the host’s genetic background contributes to cancer immunity and rare variants (Minor Allele Frequency, MAF < 1%) that are functionally deleterious have large effect size than common variants. To unveil the inherited casual variants which could influence anti-tumor immune response we performed Whole Exome Sequencing (WES) analysis in PBMCs from these 3 patients and focused on rare and predicted deleterious variants [17]. Interestingly, rare variant in *POLE* family genes was found in two patients, whereas it was observed in *ATR* gene in one patient. Moreover, rare variants in *CDC27* gene were found in all three cases. Of note, POLE, ATR and CDC27 genes are implicated in DDR response and in DNA replication stress **(**Fig. 1E**)**.

### TCGA analysis

**Fig. 2:**
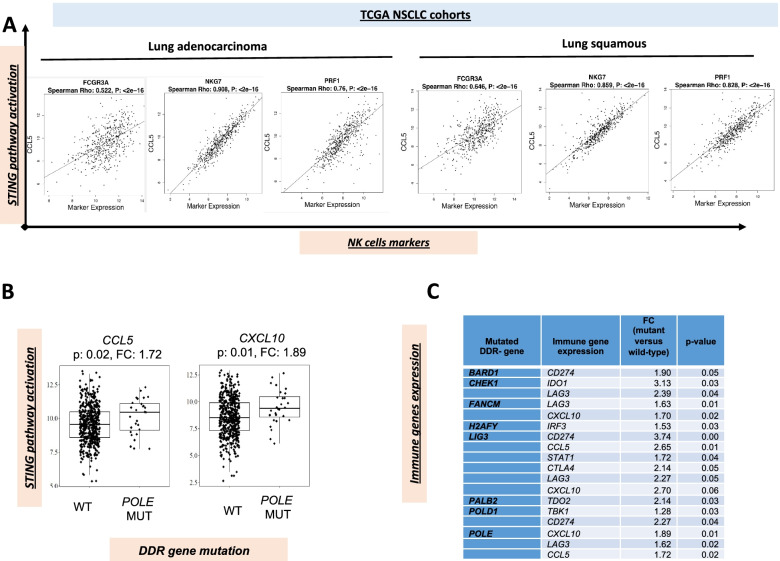
Gene expression of the STING downstream effector chemokine *CCL5* is correlated with the expression of NK cell-related genes, such as *FCGR3A* and *NKG7* (NK cell receptors) and *PRF1* (perforin, a marker of NK cell activation), with Spearman Rho> 0.5 (*p* < 0.001) in the TCGA lung adenocarcinoma cohort (*n* = 511). **B**. Comparison of differential gene expression between *POLE* mutant versus *POLE* wild-type lung adenocarcinoma (*n* = 511) or squamous carcinoma (*n* = 501) from the TCGA dataset revealed significant higher levels of STING genes (FC ≥ 1.5; *P* < 0.05 by T-test). **C**. The Table summarizes results from comparison of differential gene expression for STING/immune signatures genes between DDR mutant and DDR wild-type NSCLC: immune genes, that are correlated to immunotherapy response, with a FC ≥ 1.5 in DDR mutant samples are presented (*P* < 0.05 by T-test)

We have previously shown that activation of the STING pathway, as suggested by increased gene expression for *CCL5* and *CXCL10*, two specific STING effector chemokines, identifies a subgroup of immune checkpoint inhibitor responsive NSCLC patients [11]. Here, we have investigated a potential connection between STING pathway and NK cell activation, by correlating gene expression of *CCL5* and *CXCL10* with the expression of NK related genes in the TCGA NSCLC dataset. In this respect, *CCL5* gene expression highly correlated with *PRF1*, which encodes for perforin, a marker of NK cell activation (Spearman Rho = 0.76 and 0.828 in lung adeno-carcinoma and squamous, respectively; *p* < 0.001) and with NK cell receptor *NKG7* (Spearman Rho = 0.908 and 0.859 in lung adeno-carcinoma and squamous, respectively; *p* < 0.001); a moderate correlation was detected for the NK receptor *FCGR3A* (Spearman Rho> 0.5; *p* < 0.001) **(**Fig. 2A). Similar results were obtained for *CXCL10* (Spearman Rho> 0.5; *p* < 0.001, data not shown). The presence of DDR gene mutations have also been correlated to expression of specific immune-active transcriptomic signatures in multiple tumor types [11]. *POLE* mutations have been correlated with response to immune checkpoint inhibitors [18]. Preclinical studies have also shown that DDR inhibitors could mediate activation of innate immune pathways [10, 12]. Therefore, we next investigated the TCGA NSCLC dataset for associations between STING pathway genes and DDR gene mutations. As shown in Fig. 2B, differential gene expression between *POLE* mutant and *POLE* wild-type tumors was assessed. The STING activated genes, *CCL5* and *CXCL10,* were significantly higher in *POLE* mutant tumors (FC = 1.72; *P* = 0.02 and FC = 1.89; *P* = 0.01, respectively); thus, suggesting that *POLE* mutation could induce intrinsic STING activation. To further extend this observation, we also compared the differential expression of STING/immune signature genes, previously [11] correlated to immune-responsiveness, between DDR-mutant and DDR wild-type NSCLC samples from the TCGA lung adenocarcinoma cohort. DDR-mutant NSCLC were defined according the presence of mutation in one of DDR genes of a curated list (for the DDR gene list, see Supplementary Table 1). Of interest, the presence of mutation in at least one DDR gene, including a variety of tumors with potential heterogenous genomic landscape, occurs in NSCLC displaying features of immune-responsiveness, as indicated by gene expression of immune-genes, as listed in Fig. 2C. (Fig. 2C) [11].

### Anti-tumor activity and immune effects of cetuximab plus avelumab treatment in NSCLC patient-derived ex vivo 3D cultures

**Fig. 3:**
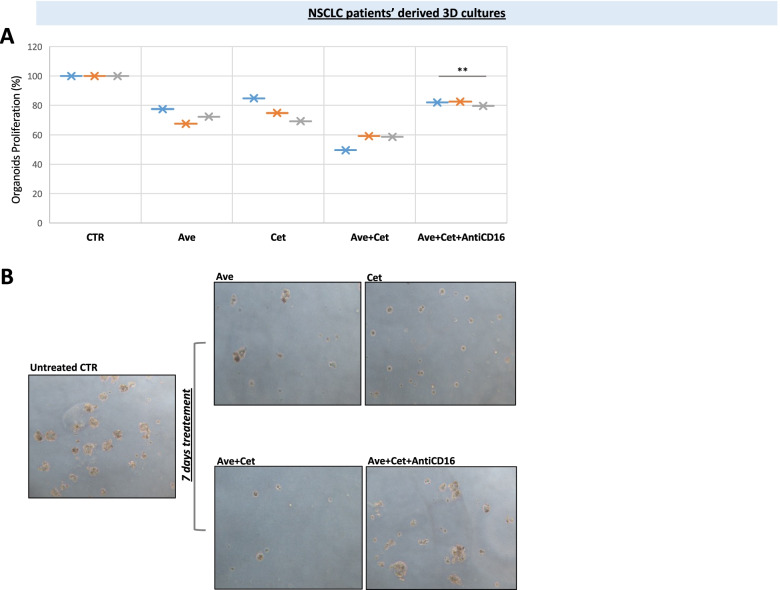
Anti-tumor effect of cetuximab plus avelumab in NSCLC patient-derived ex vivo 3D spheroid cultures. **A**. 3D spheroid cultures were derived from the 3 NSCLC patient tumor samples and treated in vitro with avelumab, cetuximab or their combination for 7 days. Treatments induced additive effect in terms of anti-tumor growth inhibition (15–30% when used as single agents, 40–50% when used in combination), as evaluated by MTS assay. This effect was partially reverted adding an anti-CD16 mAb, that neutralizes NK cells. (chi-square: 37,503, *p* < 0.05). **B**. Exemplificative images from the experiment presented in A

In vitro spheroids, that were obtained from the 3 NSCLC patients tumor samples, as previously described [15, 16], were treated with cetuximab plus avelumab for 7 days. As shown in Fig. 3A, both single agent cetuximab and avelumab determined approximately 15 to 30% growth inhibition in all cases. An additive anti-tumor activity (40 to 50% growth inhibition) was observed with the combined treatment (chi-square: 37,503, *p* < 0.05). The reduction in spheroid number and viability, that was caused by cetuximab plus avelumab treatment, was partially reverted by combined treatment with an anti-CD16 blocking mAb, that inhibited NK cell activation; thus, suggesting that NK cells are involved in the mechanism(s) of cytotoxicity of cetuximab plus avelumab. Figure 3B shows exemplificative qualitative images from each treatment point.

**Fig. 4:**
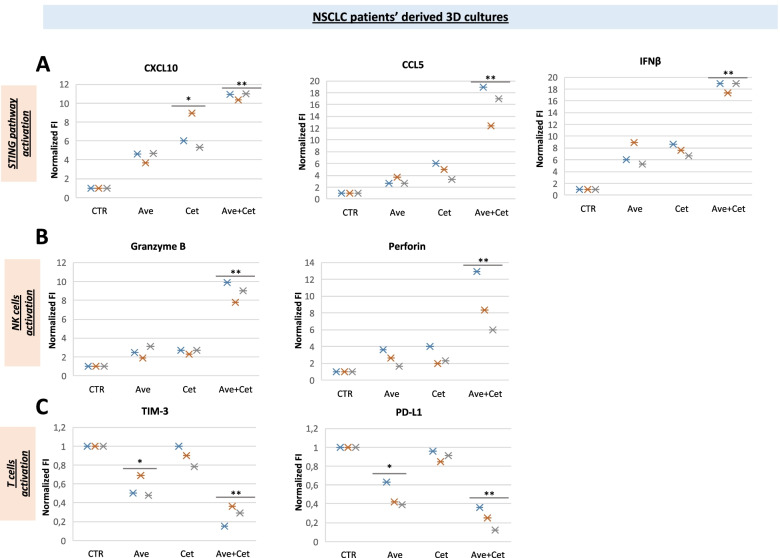
Immune-modulatory effects of cetuximab plus avelumab in NSCLC patient-derived ex vivo 3D spheroid cultures. Cetuximab plus avelumab treatment for 5 days induced 10- to 20-fold increase in gene expression, as compared to untreated control samples, for the two STING downstream effector chemokines, *CCL5* and *CXCL10,* and for *IFNβ* gene expression **(A),** and for biomarkers of NK cell activation, such as *Granzyme B* and *Perforin***(B).** Conversely, decreased levels of *PD-L1* and *TIM-3* gene expression were observed following cetuximab plus avelumab treatment, suggesting CD8+ T cell activation **(C)**. P-values were calculated by ANOVA test. **p < 0.01, *p < 0.05

To further assess potential effects of treatment with cetuximab and/or avelumab on cytokines that could be involved in NK cell activation, their gene expression was measured by quantitative PCR of RNA, that was obtained from in vitro 3D spheroid cultures. As shown in Fig. 4A, following cetuximab plus avelumab treatment a significant 10- and 20-fold increase was detected for two STING downstream effector chemokines, *CCL5* and *CXCL10*, respectively, as compared to untreated control samples. Moreover, a 20-fold increase in *IFNβ* gene expression was observed (*p* < 0.01). Similarly, a 10-fold increase in the gene expression for biomarkers of NK activation, such as of *Granzyme B* and *Perforin*, was induced by cetuximab plus avelumab treatment, as compared to untreated control samples (Fig. 4B) (*p* < 0.01). In parallel, decreased levels of *PD-L1* and *TIM-3* gene expression were found (up to 60% of decrease with combination, *p* < 0.01) suggesting also CD8+ T cell activation, as part of the anti-tumor immune response, that was induced by cetuximab plus avelumab treatment (Fig. 4C).

## Discussion

The proof-of-concept CAVE-Lung trial has recently provided the first evidence of clinical activity for cetuximab plus avelumab in pretreated NSCLC patients [5]. Among the patients enrolled in the CAVE-Lung trial, 3 patients had previously received an anti-PD-1 mAb therapy, experiencing PD after initial clinical response. If confirmed in larger clinical trials, these findings may represent a novel therapeutic approach of immunotherapy rechallenge in NSCLC patients with an anti-PD-L1 drug, avelumab, in combination with and anti-EGFR drug, cetuximab, after progression to anti-PD-1 mAbs. The combination of cetuximab plus avelumab has been also recently tested with promising clinical activity in *RAS/BRAF* wild type chemo-refractory metastatic colorectal cancer patients [19].

EGFR plays a relevant biologic role in the process of lung cancer development and progression. Preclinical data suggest that EGFR inhibition by cetuximab is accompanied by several potentially relevant immune effects, including reduction in PD-L1 expression and induction of tumor cell recognition via major histocompatibility complex (MHC) [20]. These immune-modulatory effects of cetuximab further support its potential therapeutic use in combination with immune checkpoint inhibitors in NSCLC patients.

We have previously provided evidence that the clinical benefit of cetuximab plus avelumab treatment in NSCLC patients within the CAVE-Lung trial was accompanied by NK cell activation. Here, we show that NK cell activation was prolonged during the treatment time in all 3 patients with long-term response. Clinical response to cetuximab plus avelumab treatment was accompanied by sustained activation of both NK cells (increase in CD107A+ cells) and of T cells (decrease in immune-suppressive checkpoints PD-L1+ and TIM-3+ cells). These effects were reverted at time of progression, thus suggesting that TIM3 itself may represent a mechanism of escape to this combination and a potential therapeutic target to investigate in future studies.

A main challenge in cancer therapy is to improve the clinical efficacy of immune checkpoint inhibitors in appropriately selected patients. In this scenario, there is a need for novel combinations that include drugs, that are able to modulate positively the immune microenvironment [10–13, 15]. Currently, PD-L1 expression is the only validated biomarker for patient selection for immunotherapy in NSCLC patients. It has been suggested that features of immune “hot-tumors” and of innate immunity activation, as shown, for example, by the STING pathway activation, are associated with immune responsiveness [11]. Functional features of cancer cells as well as of host immune cells, that derive from somatic and/or from germline gene mutations, could influence the immune system activation and functions [17]. Here, we report both somatic and germline mutations of genes related to DDR genes, that have been potentially implicated in the process of immune activation in all 3 patients [11, 17]. DDR gene mutations could affect both innate and adaptive immunity [21–23]. On one side, they may induce high intrinsic DNA damage and consequent high STING pathway activation [21]. On the other side, DDR mutations may sustain high tumor mutational burden and neo-antigen formation; thus, rendering cancer cells more accessible for T cell cytotoxicity [21–23]. We speculate that the presence of DDR gene mutations may be a favorable genomic substrate for anti-tumor immune response and deserve further explorations in clinical trials with novel immunotherapy combinations.

Presence of *STK11/TP53* concomitant mutations defines a specific subgroup of NSCLC with high STING pathway expression [11]. Here, by assessing the TCGA NSCLC dataset, we show that the presence of DDR mutations correlates with higher expression of STING-related genes, that, in turn, significantly correlates with biomarkers of NK cell activation. Therefore, we hypothesize that the presence of DDR family gene mutations may be a relevant functional biomarker for the anti-tumor activity of cetuximab plus avelumab. In this respect, in vitro treatment with cetuximab plus avelumab of ex vivo-derived 3D spheroid cultures from these 3 patients confirmed concomitant STING pathway and NK cell activation in parallel with anti-tumor activity.

## Conclusions

In conclusion, cetuximab plus avelumab may represent a potential novel strategy of immunotherapy combination in selected NSCLC patients. In particular, the intrinsic properties of these two IgG1 mAbs could render suitable their combination, whose anti-cancer mechanism of action is, in part, dependent on innate immunity, including NK cell-driven ADCC and STING pathway activation, as a rechallenge strategy in NSCLC patients with DDR somatic and/or germline mutations. This hypothesis requires further clinical investigation and validation.

## Supplementary Information

**Additional file 1.**

**Additional file 2.**

**Additional file 3.**

**Additional file 4.**

## Data Availability

All relevant data used for the present study are included in the manuscript.

## Abbreviations

CAVE-Lung: Cetuximab-Avelumab-Lung
mAb: Monoclonal Antibodies
NSCLC: Non-Small Cell Lung Cancer
NK: Natural Killer
ADCC: Antibody-Dependent Cell Cytotoxicity
PFS: Progression Free Survival
PBMC: Peripheral Blood Mononuclear Cells
DDR: DNA damage response
TCGA: The Cancer Genome Atlas
PD-1/PD-L1: Programmed-Death−/PD-Ligand 1
STING: Stimulator Of Interferon Genes
IFN: Interferon
Ldh: Lactate Dehydrogenase
3d: Three Dimensional
Pr: Partial Response
Sd: Stable Disease
MHC: Major Histocompatibility Complex
PCR: Polymerase chain reaction
RT-qPCR: Real Time Quantitative PCR)
WES: Whole Exome Sequencing
MTS: (3-(4,5-dimethylthiazol-2-yl)-5-(3-carboxymethoxyphenyl)-2-(4-sulfophenyl)-2H-tetrazolium)

## References

[1] Yang Y (2015). Cancer immunotherapy: harnessing the immune system to battle cancer. J Clin Invest.

[2] Ottaviano M, Curvietto M, Rescigno P, Tortora M, Palmieri G, Giannarelli D (2020). Impact of COVID-19 outbreak on cancer immunotherapy in Italy: a survey of young oncologists. J Immunother Cancer.

[3] Lobefaro R, Viscardi G, Di Liello R, Massa G, Iacovino ML, Sparano F (2021). Immunotherapy in advanced non-small cell lung Cancer patients with poor performance status: the role of clinical-pathological variables and inflammatory biomarkers. Lung Cancer.

[4] Insa A, Martín-Martorell P, Di Liello R, Fasano M, Martini G, Napolitano S (2021). Which treatment after first line therapy in NSCLC patients without genetic alterations in the era of immunotherapy?. Crit Rev Oncol Hematol.

[5] Fasano M, Della Corte CM, Di Liello R, Barra G, Sparano F, Viscardi G (2020). Induction of natural killer antibody-dependent cell cytotoxicity and of clinical activity of cetuximab plus avelumab in non-small cell lung cancer. ESMO Open.

[6] Park K, Özgüroğlu M, Vansteenkiste J, Spigel D, Yang JCH, Ishii H (2021). Avelumab versus docetaxel in patients with platinum-Treated advanced NSCLC: 2-year follow-up from the JAVELIN lung 200 phase 3 trial. J Thorac Oncol.

[7] Pirker R, Pereira JR, Szczesna A, von Pawel J, Krzakowski M, Ramlau R (2009). Cetuximab plus chemotherapy in patients with advanced non-small-celllungcancer (FLEX): an open-label randomisedphase III trial. Lancet.

[8] Lynch TJ, Patel T, Dreisbach L, McCleod M, Heim WJ, Hermann RC (2010). Cetuximab and first-line taxane/carboplatino chemotherapy in advanced non-small-cell lung cancer: results of the randomized multicenter phase III trial BMS099. J Clin Oncol.

[9] Ochoa MC, Minute L, Rodriguez I, Garasa S, Perez-Ruiz E, Inogés S (2017). Antibody-dependent cell cytotoxicity: immunotherapy strategies enhancing effector NK cells. Immunol Cell Biol.

[10] Sen T, Rodriguez BL, Chen L, Corte CMD, Morikawa N, Fujimoto J (2019). Targeting DNA damage response promotes antitumor immunity through STING-mediated T-cell activation in small cell lung Cancer. Cancer Discov.

[11] Della Corte CM, Sen T, Gay CM, Ramkumar K, Diao L, Cardnell RJ (2020). STING pathway expression identifies NSCLC with an immune-responsive phenotype. J Thorac Oncol.

[12] Sen T, Della Corte CM, Milutinovic S, Cardnell RJ, Diao L, Ramkumar K (2019). Combination treatment of the Oral CHK1 inhibitor, SRA737, and low-dose gemcitabine enhances the effect of programmed death ligand 1 blockade by modulating the immune microenvironment in SCLC. J Thorac Oncol.

[13] Deng L, Liang H, Xu M, Yang X, Burnette B, Arina A (2014). STING-dependent cytosolic DNA sensing promotes radiation-induced type I interferon-dependent antitumor immunity in immunogenic tumors. Immunity.

[14] Baysal H, De Pauw I, Zaryouh H, Peeters M, Vermorken JB, Lardon F (2021). The right partner in crime: unlocking the potential of the anti-EGFR antibody Cetuximab via combination with natural killer cell chartering immunotherapeutic strategies. Front Immunol.

[15] Della Corte CM, Barra G, Ciaramella V, Di Liello R, Vicidomini G, Zappavigna S (2019). Antitumor activity of dual blockade of PD-L1 and MEK in NSCLC patients derived three-dimensional spheroid cultures. J Exp Clin Cancer Res.

[16] Di Liello R, Ciaramella V, Barra G, Venditti M, Della Corte CM, Papaccio F (2019). Ex vivo lung cancer spheroids resemble treatment response of a patient with NSCLC to chemotherapy and immunotherapy: case report and translational study. ESMO Open.

[17] Sayaman RW, Saad M, Thorsson V, Hu D, Hendrickx W, Roelands J (2021). Germline genetic contribution to the immune landscape of cancer. Immunity.

[18] Wang F, Zhao Q, Wang YN, Jin Y, He MM, Liu ZX (2019). Evaluation of POLE and POLD1 mutations as biomarkers for immunotherapy outcomes across multiple Cancer types. JAMA Oncol.

[19] Martinelli E, Martini G, Famiglietti V, Troiani T, Napolitano S, Pietrantonio F (2021). Cetuximab Rechallenge plus Avelumab in pretreated patients with RAS wild-type metastatic colorectal Cancer: the phase 2 single-arm clinical CAVE trial. JAMA Oncol.

[20] Pollack BP, Sapkota B, Cartee TV (2011). Epidermal growth factor receptor inhibition augments the expression of MHC class I and II genes. Clin Cancer Res.

[21] Reisländer T, Groelly FJ, Tarsounas M (2020). DNA damage and Cancer immunotherapy: a STING in the tale. Mol Cell.

[22] Teo MY, Seier K, Ostrovnaya I, Regazzi AM, Regazzi AM, Kania BE, Moran MM (2018). Alterations in DNA damage response and repair genes as potential marker of clinical benefit from PD-1/PD-L1 blockade in advanced urothelial cancers. J Clin Oncol.

[23] Xiong A, Nie W, Zhou Y, Li C, Gu K, Zhang D (2021). Comutations in DDR pathways predict Atezolizumab response in non-small cell lung Cancer patients. Front Immunol.

