# Supplementary material for: Anti-tumor activity of cetuximab plus avelumab in non-small cell lung cancer patients involves innate immunity activation: findings from the CAVE-Lung trial
Source: J Exp Clin Cancer Res. 2022 Mar 26;41:109. doi: 10.1186/s13046-022-02332-2 (PMC8962159; doi:10.1186/s13046-022-02332-2)
Supplement: Supplementary file 1 — Additional file 1. [file 13046_2022_2332_MOESM1_ESM.docx]

Supplemental Table 1

| Gene | Primer sequence |
| --- | --- |
| *PD-L1* | FW: CTGCACTTTTAGGAGATTAGATCCTG  RV:TGGGATGACCAATTCAGCTGTA |
| *TIM-3* | FW: TACTGCCGGATCCAAAT  RV:TGACCTTGGCTGGTTTGATG |
| *IFN-Β* | FW: GGAAAAGCAAGAGGAAAGATTGAC  RV: CCACCATCCAGGCGTAGC |
| *CCL5* | FW: CCAGCAGTCGTCTTTGTCAC  RV: CTCTGGGTTGGCACACACTT |
| *CXCL10* | FW: GAGTGTGAAGGGCATGGCTA  RV: ATGCAAAGACAGCGTCCTCT |
| *Granzyme B* | FW: GCTCACTGTTGGGGAAGCTC  RV: GCTGCACAGCTCTGGTC |
| *Perforin* | FW: AGTGCCGCTTCTACAGTTTC  RV: GGTGCCGTAGTTGGAGATAAG |

|  |
| --- |
|  |
|  |
|  |
