# Supplementary material for: Anti-tumor activity of cetuximab plus avelumab in non-small cell lung cancer patients involves innate immunity activation: findings from the CAVE-Lung trial
Source: J Exp Clin Cancer Res. 2022 Mar 26;41:109. doi: 10.1186/s13046-022-02332-2 (PMC8962159; doi:10.1186/s13046-022-02332-2)
Supplement: Supplementary file 4 — Additional file 4. [file 13046_2022_2332_MOESM4_ESM.pdf]

Supplemental Figure 1

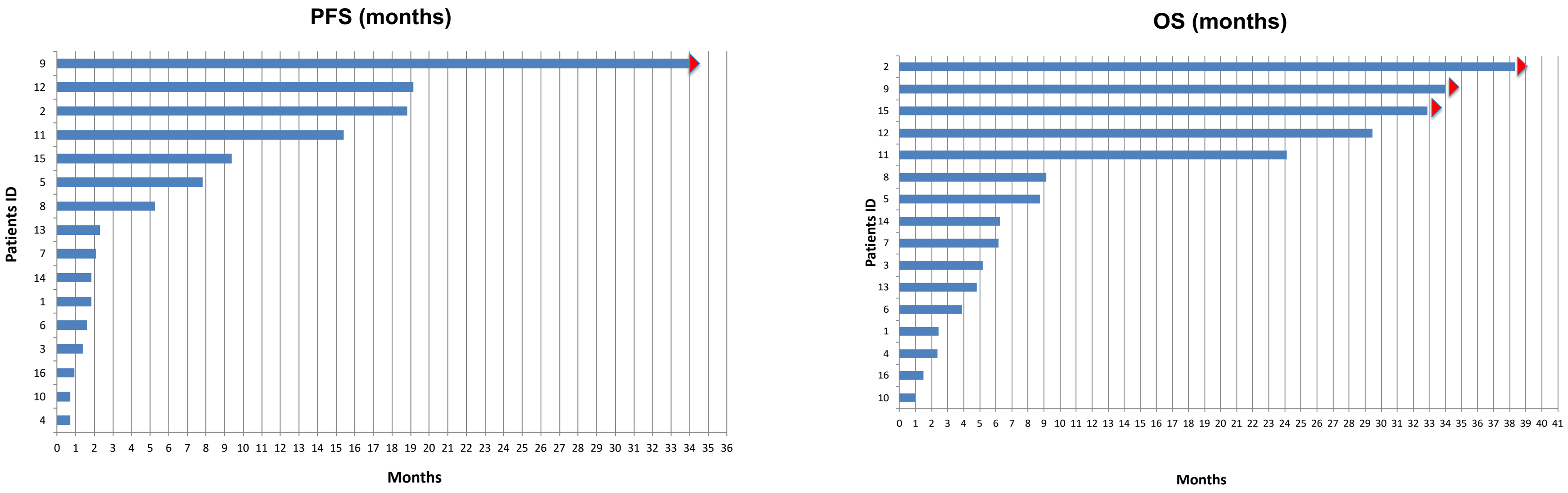

Updated PFS and OS of patients enrolled in CAVE-LUNG trial at data cut-off of November, 30th, 2021
